# Royal Chest Hospital

**Published:** 1919-03-29

**Authors:** 


### HOSPITAL ANNUAL MEETINGS.

### Royal Chest Hospital.

### HOW TO PREVENT STATE CONTROL OF VOLUNTARY HOSPITALS.

AT the 105th annual Court of Governors of the Royal Hospital for Diseases of the Chest, City Road, held last week at the Mansion House, by permission of the Lord Mayor (Sir Horace Marshall), who occupied the chair, it was unanimously decided to change the name of the institution to the "Royal Chest Hospital," and to affirm as its object "the relief of poor persons suffering from diseases of the chest in any of its various forms, including all affections of the heart and lungs."

The Lord Mayor, who is one of the vice-presidents of the hospital, said that since the arrangement was entered into with the military authorities in July of 1915 to set apart the whole of the bed accommodation at the institution for the care of sick and wounded soldiers, over 2,200 had been admitted, 793 being received last year. Although the arrangement was still in force it was hoped that very shortly the wards would again be open for the reception of civilian patients. Out-patients had increased during the past few years, new cases in 1918 numbering 2,824, and exceeding those for the preceding twelve months by 64. New cases attended to at the Tuberculosis Dispensary were 224 more than in 1917, when the number was 2,042.

Mr. Stuart De La Rue (chairman of the council of management) emphasised the greatly increasing work of the dispensary, stating that whereas in 1916 the attendances were under 8,000 last year they exceeded 18,300—an increase of over 10,300 in two years. Visits paid to homes during 1918 totalled 6,420, just double the number recorded in 1916, and over 800 more than in 1917. Speaking of the coming Ministry of Health in its relation to voluntary hospitals, Mr. De La Rue said that the establishment of the new Department would, he thought, mean that larger sums would be raised for and expended on hospitals than in the past. What he wanted to know, however, was what effect the Ministry and its actions would have on the present hospital authorities, who must, he urged, increase the efficiency of their various managements to such an extent as to render it out of the question for the Government to substitute them for machine-run hospitals. Mr. De la Rue stated that in his opinion the greatest asset of a nation lay in the physical condition of its manhood, and that the necessity for finding recruits for the Army had drawn a large measure of public attention to this fact. The capital wealth of the nation as represented by its material assets was estimated at £13,000,000,000, and even if so low a figure as £300 per head of the population were taken as the value of its living assets, a sum equal to that of the capitalised material wealth was arrived at. Capitalists could not consider 1 per cent. an excessive figure to pay for the upkeep and maintenance of their property, and if anything like such a sum were available for hospital and medical work for the country a great deal could be done by the £135,000,000 so accruing. It was significant that only a sum of £5,000,000 per annum is spent by the Metropolitan hospitals. He made an earnest appeal for funds, towards which the Lord Mayor contributed £50. Income last year was only £16,375, as compared with £28,442 in 1917—a decrease of £12,067—while the expenditure amounted to £14,409, and was £210 higher than a year previously.

### Elizabeth Garrett Anderson Hospital.

MRS. HENRY FAWCETT presided over the annual Court, when it was stated that the number of in-patients admitted last year was 1,189, comprising 797 surgical cases, 280 medical, 42 maternity, 35 infant, and 35 special cases. There were in the same period 7,988 new out-patients, whose attendances totalled 34,166. Income amounted to £13,606, and practically equalled the expenditure, there being a difference of only 12s. 8d. Both donations and subscriptions were less than in 1917—£1,744, as against £2,743—but legacies received exceeded £4,000. The endowment fund of the hospital now stands at £26,241, of which £22,000 has been invested in 5 per cent. War Loan, assuring to the hospital an additional yearly income of over £1,000. A further £13,730 has been promised, and is in course of collection, thus leaving about £11,000 to complete the desired £50,000.

### Great Northern Central Hospital.

THE MARQUIS OF NORTHAMPTON presided over the annual Court held at Holloway Road, N., when it was reported that 790 wounded soldiers passed through the wards last year. In-patients in 1918 numbered 2,834—a slightly smaller number than during the preceding year—while out-patients totalled 24,320, with 123,487 attendances, as compared with 21,669 and 104,667 attendances in 1917. Income had increased from £43,434 in that year to £51,004 during the period under review, while expenditure had risen from £41,726 to £49,002. A scheme is on foot to extend the hospital premises and provide a nurses' home at a cost of between £90,000 and £100,000 as a memorial to the men of North London who have fallen in the war. The nurses' home, which it is estimated will cost £25,000, will be the first of the proposed additions to be made.

### Samaritan Free Hospital.

THE Rev. Dr. Oxford, presided over the annual Court of Governors. Despite an increase in expenditure last year of nearly £1,000 there was, it was reported, a deficit of only £55 as compared with one of £1,179 at the end of 1917. Income, including £3,686 received as legacies, had totalled £9,996, and exceeded that of the previous year by some £2,000. The number of patients admitted fell short by 75 of those received in 1917, when a record number was cared for. During the year the Convalescent Home at Amersham had received 135 patients, who were restored to health after severe operations, many of them for cancer. On the year's working there was a deficit of £109, and an earnest appeal is made for funds to substitute the present home containing ten beds for a larger one capable of taking twenty. During the year a gift of £2,000 in National War Bonds had been received from Mr. C. F. Denny, the administrator of the estate of the late Mr. H. F. Bailey, for naming two beds in the cancer ward.

### West Kent General Hospital.

AT the annual meeting at Maidstone the report read stated that the number of in-patients had been 518, the daily average being 46, out-patients numbered 3,156, casualties and emergencies 1,570, dental attendances 958, important operations 288, and minor operations 220. The year ended with a credit balance, though the ordinary expenditure had increased by £793. Mr. George Marsham announced with regret the resignation of the matron, Miss Morton, who had filled the position since 1915. The committee had decided to recognise her services by giving her three months' salary instead of the one month due to her. Mr. Marsham added that the hospital was too small—there were sixty-five persons on the waiting-list—and it required bringing up to date. The institution was not worthy of the county town of Kent. It was suggested that the State would take over the hospitals, but he could find nothing in the Ministry of Health Bill to warrant that view.

### Hospital for the Paralysed.

AT the annual meeting, Sir Frederick Macmillan in the chair, it was reported that the expenditure of the hospital and its Finchley branch had increased from £19,108 in 1914 to £30,230 in 1918. Throughout the war seventy beds had been provided for soldiers suffering from nerve injuries and affections, and this work was being followed up by special provision, in three branch hospitals, for discharged men, in connection with the Ministry of Pensions. The hospital and its branches suffered acutely in the influenza epidemic, and the Board Room was utilised as a special isolation ward.

